# Poly[bis­(*O*-ethyl­hydroxy­laminium) [di-μ-chlorido-di­chlorido­cadmate(II)]]

**DOI:** 10.1107/S2414314623008982

**Published:** 2023-10-19

**Authors:** Yu-Qiao Tong, Zi-Qiong Lei, Bo Huang

**Affiliations:** a Faculty of Chemistry and Chemical Engineering, Yunnan Normal University, Kunming, 650050, People’s Republic of China; Vienna University of Technology, Austria

**Keywords:** crystal structure, *O*-ethyl­hydrox­ylammonium, organic-inorganic hybrid, layered perovskite

## Abstract

The title compound, {(CH_3_CH_2_ONH_3_)_2_[CdCl_4_]}_*n*_, consists of bilayers of organic CH_3_CH_2_ONH_3_^+^ cations and infinite [CdCl_4_]_*n*_^2*n*−^ inorganic layers. It can be described as an organic–inorganic hybrid layered perovskite. In the crystal structure, the Cd^II^ cation is situated at an inversion center and is coordinated by six chloride ions, forming a slightly distorted octa­hedral coordination polyhedron. By corner-sharing of the [CdCl_6_] octa­hedra, infinite [CdCl_4_]_*n*_^2*n*−^ inorganic layers are formed, extending parallel to (100). The inorganic layers alternate with bilayers of CH_3_CH_2_ONH_3_^+^ cations, whereby the connection of the cationic and anionic layers is achieved through N—H⋯Cl hydrogen bonds and Coulombic inter­actions.

## Abstract 2

In the crystal structure of the title organic–inorganic hybrid layered perovskite, organic bilayers alternate with [CdCl_4_]_*n*_^2*n*−^ inorganic layers along [100].

## Graphical abstract

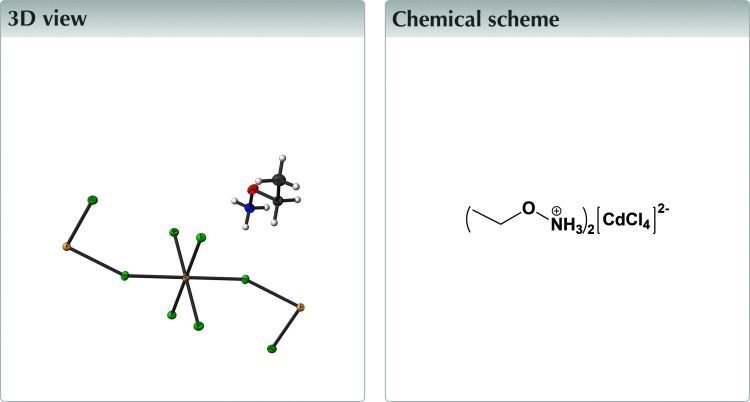

## Structure description

As a class of mol­ecular materials with the advantages of low density, mechanical flexibility, and being easy to process into thin films, organic–inorganic hybrid layered perovskite compounds have attracted a lot of attention on account of their outstanding ferroelectric, multiferroic, and semiconducting performance (Huang *et al.*, 2018 ▸). However, it is hard to predict and design advanced materials with specific performance. One reason is the lack of understanding as to why a particular crystal structure forms (Sun *et al.*, 2020 ▸). In this regard, it is fundamentally important to search for and study new examples of such organic–inorganic hybrid layered perovskite compounds (Yang *et al.*, 2022 ▸). Herein, we report the synthesis and crystal structure of the title compound, (CH_3_CH_2_ONH_3_)^+^_2_[CdCl_4_]^2–^, based on *O*-ethyl­hydroxyl­ammonium cations and tetra­chlorido­cadmate anions.

**Figure 1:**
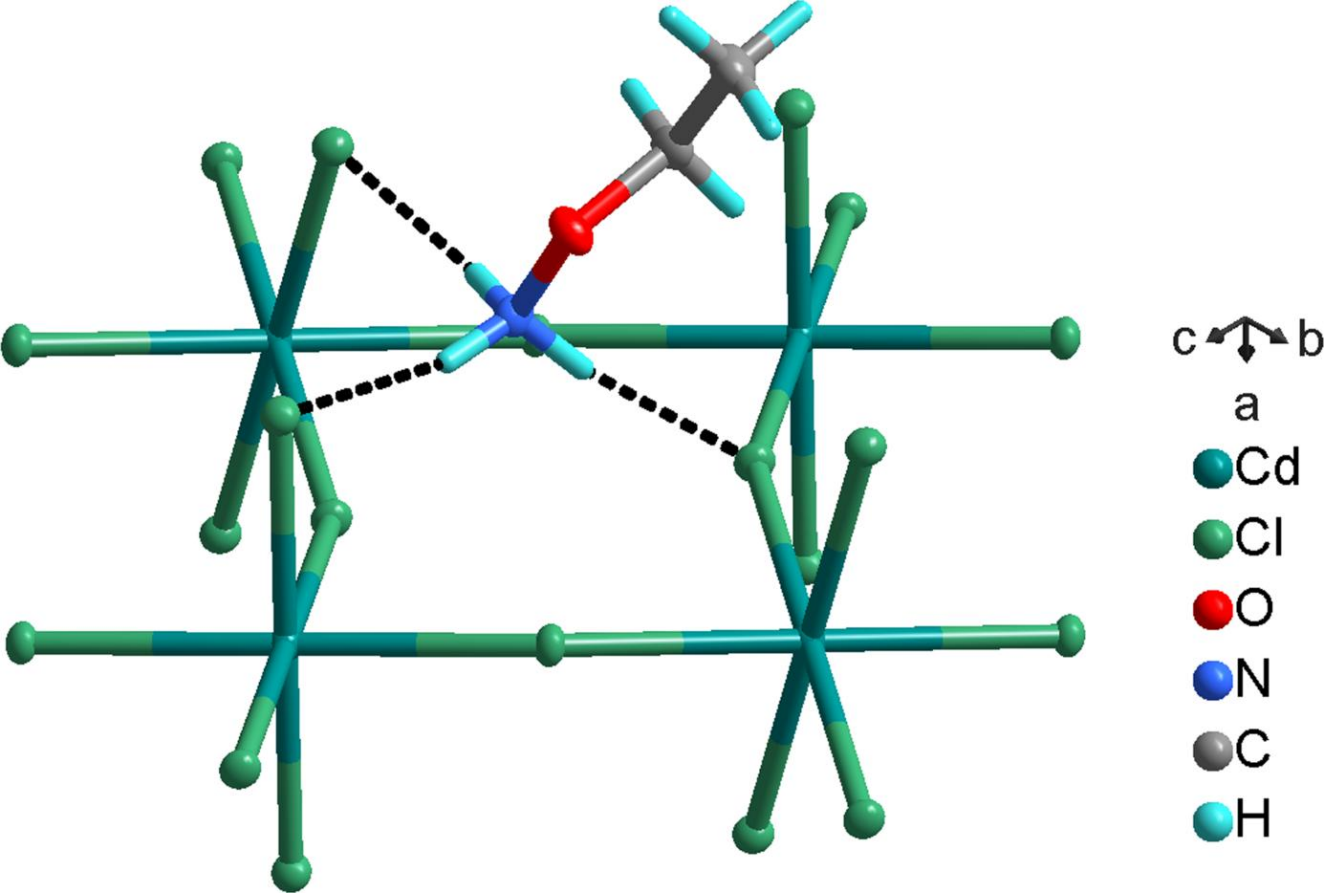
A part of the crystal structure of the title compound showing the coordination around the Cd^II^ cation, and the N—H⋯Cl hydrogen-bonding inter­actions (dotted lines) between the cation and the anionic layer. Displacement ellipsoids are drawn at the 50% probability level.

The asymmetric unit contains one Cd^II^ cation, two chloride anions and one *O*-ethyl­hydroxyl­ammonium cation. The Cd^II^ cation is situated at an inversion center (Wyckoff site *b*) and is distorted octa­hedrally coordinated by six chloride anions (Fig. 1 ▸). Two medium and two long equatorial Cd—Cl1 bonds [2.6798 (5) and 2.7416 (5) Å, respectively], and two shorter axial Cd—Cl2 bonds [2.5384 (5) Å] are present.

**Figure 2:**
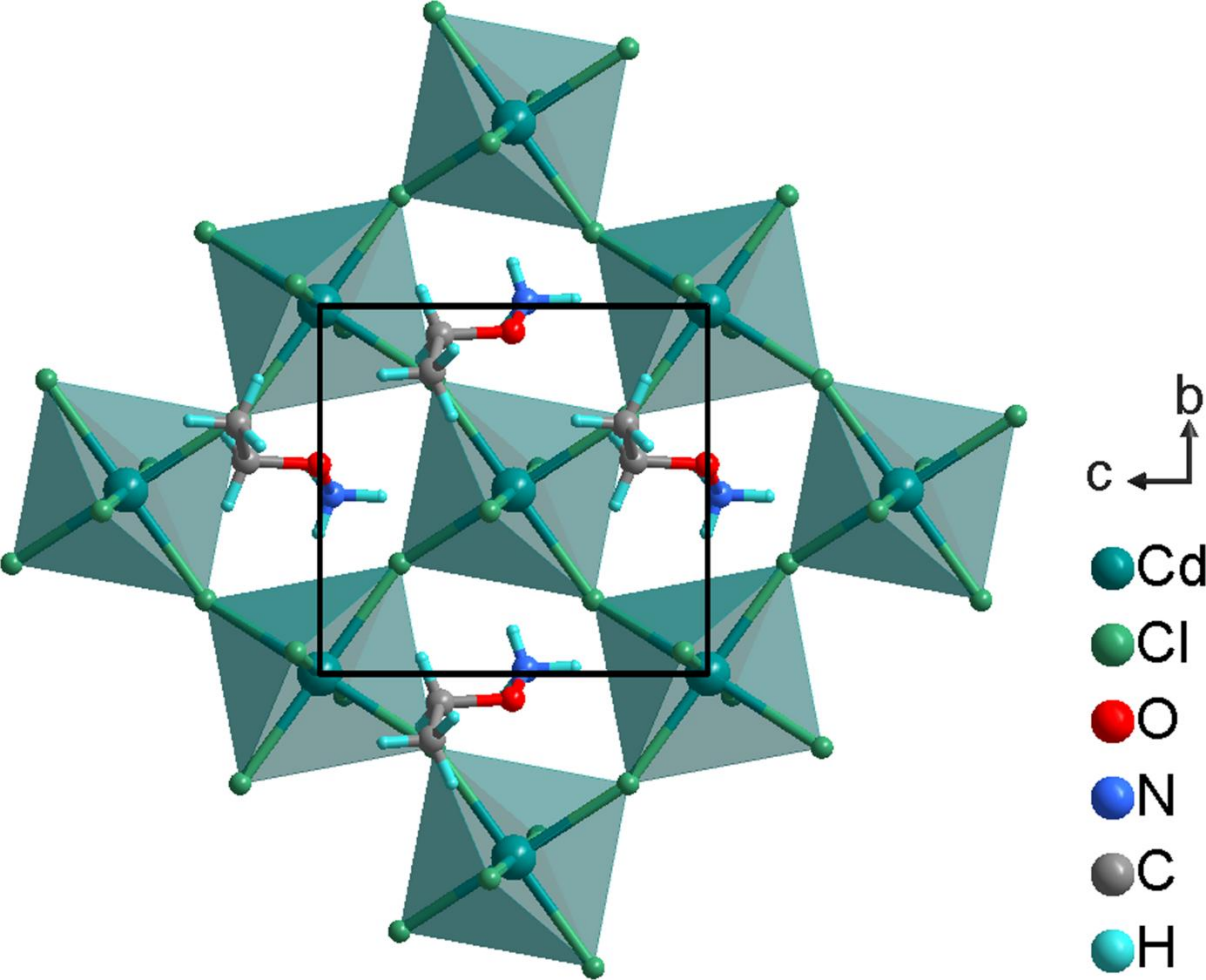
The organic cations and the anionic [CdCl_4_]^2–^ layer (polyhedral representation) in the title compound, in a view along [100].

**Figure 3:**
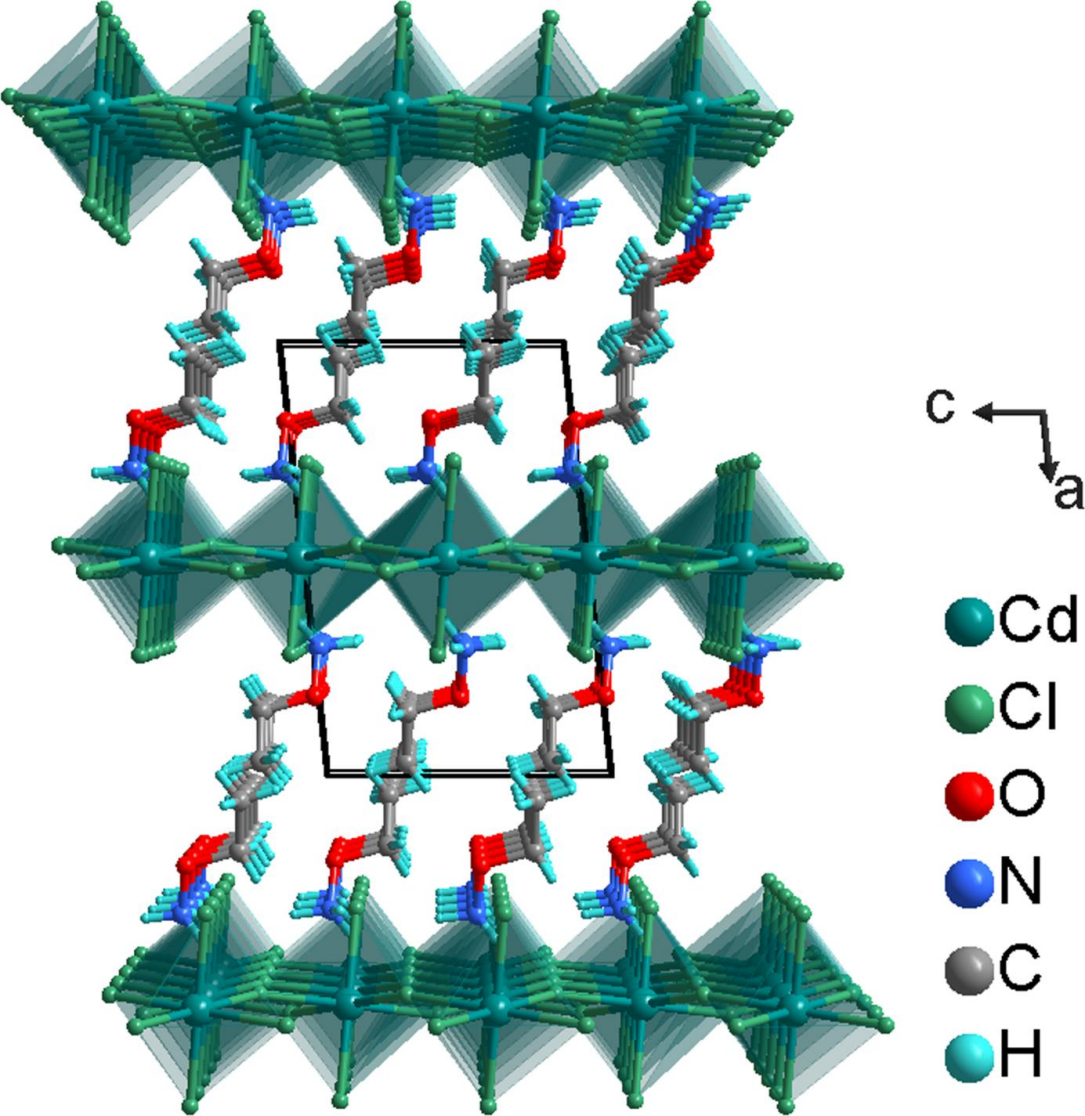
The stacking of (100) layers of organic cations in bilayers and anionic [CdCl_4_]^2–^ layers (polyhedral representation) along [100], in a view along [010].

The structure of the title compound can be described as an organic–inorganic hybrid layered perovskite with general formula *A*_2_ *MX*_4_ (*A* = monovalent organic cation, *M* = divalent metal cation, *X* = halide anion). By corner-sharing of the [CdCl_6_] octa­hedra, infinite inorganic [CdCl_4_]_*n*_^2*n*−^ layers are formed, extending parallel to (100) (Fig. 2 ▸). Neighboring inorganic layers alternate with bilayers of organic CH_3_CH_2_ONH_3_^+^ cations along [100] (Fig. 3 ▸). The CH_3_CH_2_ONH_3_^+^ cation is N—H⋯Cl hydrogen-bonded to three [CdCl_6_] octa­hedra with two hydrogen bonds to the axial Cl ligand, and one hydrogen bond to an equatorial ligand (Fig. 1 ▸, Table 1 ▸). The cohesion between the inverted cations in the organic bilayer is achieved through van der Waals forces.

## Synthesis and crystallization

An aqueous solution (15 ml) containing stoichiometric qu­anti­ties of *O*-ethyl­hydroxyl­ammonium (5 mmol), CdCl_2_ (2.5 mmol), and hydro­chloric acid (5 mmol) was stirred for 15 min. The clear solution was allowed to stand at room temperature for slow evaporation. About one week later, colorless, plate-shaped crystals of (CH_3_CH_2_ONH_3_)_2_[CdCl_4_] were obtained in about 83% yield based on Cd.

## Refinement

Crystal data, data collection and structure refinement details are summarized in Table 2 ▸.

## Supplementary Material

Crystal structure: contains datablock(s) I. DOI: 10.1107/S2414314623008982/wm4199sup1.cif

CCDC reference: 2301057

Additional supporting information:  crystallographic information; 3D view; checkCIF report

## Figures and Tables

**Table 1:** Hydrogen-bond geometry (Å, °)

*D*—H⋯*A*	*D*—H	H⋯*A*	*D*⋯*A*	*D*—H⋯*A*
N1—H1*A*⋯Cl1	0.89	2.38	3.259 (2)	169
N1—H1*B*⋯Cl2^i^	0.89	2.31	3.1810 (18)	167
N1—H1*C*⋯Cl2^ii^	0.89	2.34	3.166 (2)	155

Symmetry codes: (i) 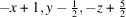 ; (ii) 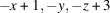 .

**Table 2:** Experimental details

Crystal data	
Chemical formula	(C_2_H_8_NO)_2_[CdCl_4_]
*M*_r_	378.39
Crystal system, space group	Monoclinic, *P*2_1_/*c*
Temperature (K)	150
*a*, *b*, *c* (Å)	11.7058 (8), 7.2365 (5), 7.6864 (5)
β (°)	96.374 (2)
*V* (Å^3^)	647.08 (8)
*Z*	2
Radiation type	Mo *K*α
μ (mm^−1^)	2.49
Crystal size (mm)	0.30 × 0.30 × 0.10

Data collection	
Diffractometer	Oxford Diffraction, Xcalibur, Eos, Gemini CCD
Absorption correction	Multi-scan (*CrysAlis PRO*; Rigaku OD, 2019 ▸)
*T*_min_, *T*_max_	0.450, 1.000
No. of measured, independent and observed [*I* > 2σ(*I*)] reflections	6115, 1462, 1401
*R*_int_	0.052
(sin θ/λ)_max_ (Å^−1^)	0.647

Refinement	
*R*[*F* ^2^ > 2σ(*F* ^2^)], *wR*(*F* ^2^), *S*	0.023, 0.054, 1.07
No. of reflections	1462
No. of parameters	63
H-atom treatment	H-atom parameters constrained
Δρ_max_, Δρ_min_ (e Å^−3^)	1.39, −0.86

Computer programs: *CrysAlis PRO* (Rigaku OD, 2019 ▸), *SHELXT* (Sheldrick, 2015 ▸), *OLEX2* (Dolomanov *et al.*, 2009 ▸), *DIAMOND* (Brandenburg, 1999 ▸) and *publCIF* (Westrip, 2010 ▸).

## full crystallographic data

### Crystal data

**Table:**

(C_2_H_8_NO)_2_[CdCl_4_]	*F*(000) = 372
*M**_r_* = 378.39	*D*_x_ = 1.942 Mg m^−^^3^
Monoclinic, *P*2_1_/*c*	Mo *K*α radiation, λ = 0.71073 Å
*a* = 11.7058 (8) Å	Cell parameters from 6460 reflections
*b* = 7.2365 (5) Å	θ = 3.3–27.5°
*c* = 7.6864 (5) Å	µ = 2.49 mm^−^^1^
β = 96.374 (2)°	*T* = 150 K
*V* = 647.08 (8) Å^3^	Plate, colourless
*Z* = 2	0.30 × 0.30 × 0.10 mm

### Data collection

**Table:**

Oxford Diffraction, Xcalibur, Eos, Gemini CCD diffractometer	1401 reflections with *I* > 2σ(*I*)
Graphite monochromator	*R*_int_ = 0.052
ω scans	θ_max_ = 27.4°, θ_min_ = 3.3°
Absorption correction: multi-scan (CrysAlisPro; Rigaku OD, 2019)	*h* = −15→15
*T*_min_ = 0.450, *T*_max_ = 1.000	*k* = −9→9
6115 measured reflections	*l* = −9→9
1462 independent reflections	

### Refinement

**Table:**

Refinement on *F*^2^	0 restraints
Least-squares matrix: full	Hydrogen site location: inferred from neighbouring sites
*R*[*F*^2^ > 2σ(*F*^2^)] = 0.023	H-atom parameters constrained
*wR*(*F*^2^) = 0.054	*w* = 1/[σ^2^(*F*_o_^2^) + (0.0147*P*)^2^ + 0.3935*P*] where *P* = (*F*_o_^2^ + 2*F*_c_^2^)/3
*S* = 1.07	(Δ/σ)_max_ = 0.001
1462 reflections	Δρ_max_ = 1.39 e Å^−^^3^
63 parameters	Δρ_min_ = −0.86 e Å^−^^3^

### Special details

**Table:**

Geometry. All esds (except the esd in the dihedral angle between two l.s. planes) are estimated using the full covariance matrix. The cell esds are taken into account individually in the estimation of esds in distances, angles and torsion angles; correlations between esds in cell parameters are only used when they are defined by crystal symmetry. An approximate (isotropic) treatment of cell esds is used for estimating esds involving l.s. planes.
Refinement. All H atoms were generated by geometrical considerations and constrained to their idealized positions.

### Fractional atomic coordinates and isotropic or equivalent isotropic displacement parameters (Å^2^)

**Table:**

	*x*	*y*	*z*	*U*_iso_*/*U*_eq_	
Cd1	0.500000	0.000000	1.000000	0.01349 (9)	
Cl1	0.47138 (4)	0.20056 (6)	1.29465 (6)	0.01647 (12)	
Cl2	0.71450 (4)	0.05786 (7)	1.06269 (6)	0.01747 (12)	
O1	0.18746 (12)	0.0684 (2)	1.49656 (18)	0.0207 (3)	
N1	0.29792 (17)	−0.0137 (2)	1.5301 (3)	0.0197 (4)	
H1A	0.348789	0.053052	1.479307	0.024*	
H1B	0.295778	−0.127949	1.486972	0.024*	
H1C	0.318415	−0.017670	1.645068	0.024*	
C1	0.15465 (18)	0.0791 (3)	1.3096 (3)	0.0221 (4)	
H1D	0.147058	−0.043642	1.258934	0.026*	
H1E	0.211707	0.147040	1.253094	0.026*	
C2	0.04102 (19)	0.1784 (4)	1.2877 (3)	0.0285 (5)	
H2A	0.014672	0.189435	1.165319	0.043*	
H2B	0.050075	0.299323	1.338706	0.043*	
H2C	−0.014190	0.109786	1.345010	0.043*	

### Atomic displacement parameters (Å^2^)

**Table:**

	*U*^11^	*U*^22^	*U*^33^	*U*^12^	*U*^13^	*U*^23^
Cd1	0.01489 (13)	0.01250 (14)	0.01301 (13)	−0.00030 (6)	0.00125 (9)	0.00052 (6)
Cl1	0.0192 (2)	0.0145 (2)	0.0160 (2)	−0.00024 (16)	0.00327 (18)	−0.00387 (16)
Cl2	0.0156 (2)	0.0190 (3)	0.0181 (2)	−0.00023 (17)	0.00301 (18)	0.00076 (18)
O1	0.0193 (7)	0.0255 (8)	0.0173 (7)	0.0078 (6)	0.0025 (6)	0.0020 (6)
N1	0.0178 (9)	0.0196 (10)	0.0215 (10)	0.0015 (6)	0.0009 (8)	0.0016 (6)
C1	0.0234 (10)	0.0272 (12)	0.0155 (10)	−0.0014 (8)	0.0016 (8)	0.0021 (8)
C2	0.0238 (11)	0.0329 (13)	0.0279 (11)	0.0026 (9)	−0.0006 (9)	0.0074 (9)

### Geometric parameters (Å, º)

**Table:**

Cd1—Cl1^i^	2.6798 (5)	N1—H1B	0.8900
Cd1—Cl1	2.7416 (5)	N1—H1C	0.8900
Cd1—Cl1^ii^	2.7416 (5)	C1—H1D	0.9700
Cd1—Cl1^iii^	2.6798 (5)	C1—H1E	0.9700
Cd1—Cl2^ii^	2.5384 (5)	C1—C2	1.505 (3)
Cd1—Cl2	2.5384 (5)	C2—H2A	0.9600
O1—N1	1.420 (2)	C2—H2B	0.9600
O1—C1	1.448 (2)	C2—H2C	0.9600
N1—H1A	0.8900		
			
Cl1^i^—Cd1—Cl1^iii^	180.0	O1—N1—H1B	109.5
Cl1^ii^—Cd1—Cl1	180.0	O1—N1—H1C	109.5
Cl1^iii^—Cd1—Cl1	87.722 (7)	H1A—N1—H1B	109.5
Cl1^iii^—Cd1—Cl1^ii^	92.278 (7)	H1A—N1—H1C	109.5
Cl1^i^—Cd1—Cl1	92.279 (7)	H1B—N1—H1C	109.5
Cl1^i^—Cd1—Cl1^ii^	87.722 (7)	O1—C1—H1D	110.6
Cl2—Cd1—Cl1^ii^	92.012 (14)	O1—C1—H1E	110.6
Cl2^ii^—Cd1—Cl1^i^	88.007 (15)	O1—C1—C2	105.70 (17)
Cl2^ii^—Cd1—Cl1^iii^	91.993 (15)	H1D—C1—H1E	108.7
Cl2^ii^—Cd1—Cl1^ii^	87.988 (14)	C2—C1—H1D	110.6
Cl2—Cd1—Cl1^iii^	88.007 (15)	C2—C1—H1E	110.6
Cl2—Cd1—Cl1^i^	91.993 (15)	C1—C2—H2A	109.5
Cl2—Cd1—Cl1	87.988 (15)	C1—C2—H2B	109.5
Cl2^ii^—Cd1—Cl1	92.012 (15)	C1—C2—H2C	109.5
Cl2—Cd1—Cl2^ii^	180.00 (2)	H2A—C2—H2B	109.5
Cd1^iv^—Cl1—Cd1	153.62 (2)	H2A—C2—H2C	109.5
N1—O1—C1	109.78 (15)	H2B—C2—H2C	109.5
O1—N1—H1A	109.5		
			
N1—O1—C1—C2	−176.40 (17)		

Symmetry codes: (i) *x*, −*y*+1/2, *z*−1/2; (ii) −*x*+1, −*y*, −*z*+2; (iii) −*x*+1, *y*−1/2, −*z*+5/2; (iv) −*x*+1, *y*+1/2, −*z*+5/2.

### Hydrogen-bond geometry (Å, º)

**Table:**

*D*—H···*A*	*D*—H	H···*A*	*D*···*A*	*D*—H···*A*
N1—H1*A*···Cl1	0.89	2.38	3.259 (2)	169
N1—H1*B*···Cl2^iii^	0.89	2.31	3.1810 (18)	167
N1—H1*C*···Cl2^v^	0.89	2.34	3.166 (2)	155

Symmetry codes: (iii) −*x*+1, *y*−1/2, −*z*+5/2; (v) −*x*+1, −*y*, −*z*+3.
